# Plasmid-based target protectors allow specific blockade of miRNA silencing activity in mammalian developmental systems

**DOI:** 10.3389/fncel.2013.00163

**Published:** 2013-09-24

**Authors:** Jennifer L. Knauss, Shan Bian, Tao Sun

**Affiliations:** ^1^Department of Cell and Developmental Biology, Weill Medical College of Cornell UniversityNew York, NY, USA; ^2^Weill Cornell Graduate School of Medical SciencesNew York, NY, USA

**Keywords:** miRNA, miR-19, Pten, mRNA protector, neural development

## Abstract

Over the past decade, microRNAs (miRNAs) have emerged as essential posttranscriptional regulators of gene expression. Though a great deal has been discovered about miRNA genomics, biogenesis, mechanisms, and functions, the challenge of attributing phenotypes of altered miRNA expression to specific targets still remains. Here, we apply the existing target protector concept of blocking miRNA action at a single binding site in the 3′untranslated region (3′UTR) of its target to a plasmid-based approach. We optimize and demonstrate target protector efficacy *in vitro*, where it blocks repression of a luciferase construct and an endogenous protein. Using the developing mouse cortex as a model, we validate that target protectors are effective *in vivo*, where protectors for the miR-19a binding sites in the *Pten* 3′UTR alter proliferation and specification of neural progenitors, phenocopying Pten ectopic expression phenotypes. Our study introduces a new tool for analyzing specific miRNA:target interactions across mammalian developmental systems, facilitating further miRNA functional discoveries.

## INTRODUCTION

MicroRNAs (miRNAs) are an extensive class of small non-coding RNAs that are critically important throughout development (Carrington and Ambros, 2003; Bartel, 2004; Alvarez-Garcia and Miska, 2005; Kloosterman and Plasterk, 2006). They regulate gene expression posttranscriptionally through incorporation into a RNA-induced silencing complex (RISC), which uses partial sequence complementarity to bind target messenger RNAs (mRNAs), usually in the 3′untranslated region (3′UTR; Lewis et al., 2003). RISC binding can lead to blocking translation or enhancing the decay of target mRNAs, the final result being downregulation of the target protein (Bartel, 2004). Hundreds of miRNAs have been identified in the genomes of mammals including rodents and humans, and each miRNA can regulate many mRNAs in a cell-type specific manner, leading to a complex network of interactions (Landgraf et al., 2007). It is estimated that between 25 and 60% of human transcripts are regulated by miRNAs (Lewis et al., 2005; Lim et al., 2005; Friedman et al., 2009).

Conditional knockout and knockdown techniques have been used to examine the roles of miRNAs; however, there can be difficulty in interpreting molecular mechanisms underlying the observed phenotypes due to the large number of potential targets of each miRNA. Groups working in model systems such as zebrafish and *Xenopus* have bypassed this obstacle through a morpholino target protector approach, using sequence complementarity to block a miRNA from binding to a specific site (Choi et al., 2007; Bonev et al., 2011; Stanton and Giraldez, 2011). However, morpholinos are not a tractable tool in mammals and their short length of activity limits their application in developmental systems.

We here have developed a plasmid-based target protector system to tease apart the physiological roles of miRNAs in mammalian systems. Previous work in our lab has shown that in the developing cortex, *miR-19a* targets *Pten* mRNA (Bian et al., 2013). In the developing mouse cortex, Pten functions to repress progenitor expansion; therefore its repression by *miR-19a* results in increased proliferation (Groszer et al., 2001; Zheng et al., 2008). Thus, the *miR-19a*:*Pten* relationship provides an ideal readout for testing *Pten* derepression through target protectors. Here, we have designed and optimized target protectors for the miR-19a binding sites in the *Pten* 3′UTR. We demonstrate that these target protectors can be electroporated *in utero* to allow functional investigation of a specific miRNA:mRNA interaction during cortical development *in vivo*. Our results provide a useful tool for investigation of long term, specific miRNA–target interactions both *in vitro* and *in vivo* using a plasmid-based target protector system.

## MATERIALS AND METHODS

### TARGET PROTECTOR DESIGN

Protectors were designed as perfectly complementary sequence covering the miR-19a binding sites in the *Pten* 3′UTR. The miRNA seed binding sequence was centered in the target protector, with complementary sequence on each side. Outside of the complementary sequence, restriction sites can be added as necessary for a cloning strategy.

For the second miR-19a binding site, target protectors with three lengths of complementarity to the *Pten* 3′UTR were designed: 22, 40, and 60 nucleotides (nt; **Figure 2A**). All of the target protectors were designed to be the same total length as the 60 nt protector and included junk sequences to increase their length as necessary, keeping the target protector in the middle of the construct. We ordered the target protectors as complementary oligonucleotides. After annealing, protectors were subcloned and inserted into the pCAGIG vector for electroporation and pCDNA3.1 for the luciferase assay.

### miR-19a EXPRESSION CONSTRUCT

The precursor hairpin sequence of miR-19a and ~100 nt of genomic sequence flanking each side of the hairpin sequence was amplified by PCR from the genomic locus of the mouse miR-17-92 cluster. Sequences of primers are as following: miR-19a: F: 5′-CAGCTCGAGCAATCCAAGTCA-3′, R: 5′-GCAGGCTCTACATCGACAC-3′. To generate the miR-19a expression construct, the miRNA fragment was inserted into pcDNA3.1 for transfection in cell lines, and pCAGIG for electroporation.

### LUCIFERASE ASSAY

pGL4.13 firefly luciferase (Promega) vector was used for making constructs containing amplified 3′UTRs of targets. pGL4.73 renilla luciferase (Promega) was used as a transfection control. Plasmid DNA was quantified by UV spectrophotometry and used for transfection in a 6:2:1 ratio (protector:miRNA:target luciferase constructs) in Neuro2a (N2a) cells using Lipofectamine 2000 (Invitrogen) according to the manufacturer’s protocol. Luciferase was activated using the Dual-Luciferase Reporter Assay kit (Promega) using the manufacturer’s protocol and read on a Victor3 1420 multilabel counter (Perkin Elmer). Results were shown as firefly luciferase activity normalized to renilla as controls.

To make the 3′UTR construct for the luciferase assay, a cDNA fragment encoding the mouse Pten 3′UTR was amplified and subcloned into the pGL4.13 luciferase vector. The first miR-19a binding site was mutated using QuikChange II Site-Directed Mutagenesis Kit (Agilent Technologies) according to manufacturer’s instructions. All the primers for cloning of targets in the 3′UTR and their mutation are listed as the following: Pten-3′UTR: F: 5′-CATCTAGAATACATCCACAGGGTTTTGACA-3′, R: 5′-TTGAAGCCCTAATCCCAACTCT-3′; Pten-3′UTR-miR-19a-mut1: 5′-CCGGGTTCACGTCCTACCCCATTACAATTGTGGCAACAGATAAGTTT-3′.

### NORTHERN BLOT ANALYSIS

Total RNA was isolated from N2a cells transfected with either the 60 nt target protector or the pcDNA3.1 empty vector using Trizol reagent (Invitrogen) according to manufacturer’s instructions. RNA samples and 0.1–2 kb RNA ladder (Invitrogen) were denatured at 70°C for 15 min and cooled on ice. Ethidium bromide was added to the RNA ladder for visualization. The DNA control sample was denatured at 95°C and cooled on ice. Samples were loaded onto a 1% formaldehyde agarose gel and separated at room temperature. After running, the ladder band locations were marked on the gel.

Samples were transferred onto a nitrocellulose membrane using a semi-dry transfer method overnight. After transfer, the ladder band locations were marked on the membrane. After cross-linking for 4 h at 80°C, the membrane was hybridized at 50°C overnight using a denatured DNA probe for the 60 nt target protector. The probe was body-labeled with digoxygenin (DIG)-labeled nucleotides using the DIG DNA Labeling Kit (Roche), following manufacturer instructions. After washing, the RNA was detected using the CDP-star chemiluminescent substrate (Roche).

### WESTERN BLOT ANALYSIS

Expression levels of Pten were analyzed by the Western blot analysis. Protein extracts were harvested by lysing N2a cells transfected with combinations miR-19a, 60 nt target protector, and empty vector with RIPA lysis buffer (150 mM NaCl, 1 mM Na_4_P_2_O_7_, 1 mM NaF, 1 mM EDTA, 1 mM PMSF, 2 mM Na_3_VO_4_, 1% NP-40, 50 mM Tris, pH 7.5) with complete^TM^ EDTA-free protease inhibitor mixture (Roche Diagnostics, Indianapolis, IN, USA). The protein samples were boiled in SDS sample buffer for 10 min before loading onto 10% Tris–glycine gels as 10 μg for each lane and transferred onto PVDF membrane (Pall Corporation, Pensacola, FL, USA). For immunoblotting, membranes were blocked with 5% (w/v) non-fat milk powder in 0.05% TBST [50 mM Tris–Cl, pH 7.5, 150 mM NaCl, with 0.05% (v/v) Tween-20] and incubated at 4°C overnight with the following primary antibodies which were diluted in 0.05% TBST with 5% non-fat milk: Pten and actin. After washing with TBST, membranes were incubated with specific HRP-conjugated secondary antibodies for 1 h at room temperature followed with extended washes with TBST. Immunoblot reactions were visualized using chemiluminescent substrate (Pierce, Rockford, IL, USA) on Kodak BioMax light films (Kodak, Rochester, NY, USA). The intensities of the bands were densitometrically quantified with the image software ImageJ.

The following antibodies were used: anti-Pten (rabbit, 1:1,000; Cell Signaling) and anti-Actin (rabbit, 1:400; Sigma).

### *IN UTERO* ELECTROPORATION

*In utero* electroporation was performed as described by Saito (2006). Briefly, electroporation was conducted at E13.5 and the brain tissues were harvested 24 h later at E14.5. Plasmid DNA was prepared using the EndoFree plasmid maxi kit (Qiagen) according to manufacturer’s instructions, and diluted to 2.5 μg/μl. DNA solution was injected into the lateral ventricle of the cerebral cortex, and electroporated with five 50-ms pulses at 35 V using an ECM830 electrosquareporator (BTX).

Wild type CD-1 mice were used for all experiments. For staging of embryos, midday of the day of vaginal-plug formation was considered E0.5; the first 24 h after birth were defined as P0. Animal use was overseen by the Animal Facility at Weill Cornell Medical College.

### TISSUE PREPARATION AND IMMUNOHISTOCHEMISTRY

Mouse brains were fixed in 4% paraformaldehyde (PFA) in phosphate-buffered saline (PBS) over night, incubated in 25–30% sucrose in PBS, embedded in OCT and stored at -80°C until use. Brains were sectioned (10–14 μm) using a cryostat. For antigen recovery, sections were incubated in heated (95–100°C) antigen recovery solution (1 mM EDTA, 5 mM Tris, pH 8.0) for 15–20 min, and cooled down for 20–30 min. Before applying antibodies, sections were blocked in 10% normal goat serum (NGS) in PBS with 0.1% Tween-20 (PBT) for 1 h. Sections were incubated with primary antibodies at 4°C overnight and visualized using goat anti-rabbit IgG-Alexa-Fluor-488 and/or goat anti-mouse IgG-Alexa-Fluor-546 (1:350, Molecular Probes) for 1.5 h at room temperature. Images were captured using a Leica digital camera under a Zeiss confocal microscope.

Primary antibodies against the following antigens were used: bromodeoxyuridine (BrdU; 1:50, DSHB), Ki67 (1:500, Abcam), Pax6 (1:200, Covance, rabbit), Tbr2 (1:500, Abcam), GFP (1:1000, Abcam, chicken), and GFP (1:1000, Rockland, rabbit).

### CELL COUNTING

Coronal sections were collected in the medial cortical region (at levels between the anterior commissure and the anterior hippocampus). At least four sections from each brain and three brains from different litters were chosen for antibody labeling. Positive cells were quantified in fixed areas of 318 μm × 318 μm and normalized to the averaged empty vector control value.

### STATISTICS

For the luciferase and Western blot assays, three independent experiments were performed. For electroporated mouse sections, at least three brains from each group were analyzed. Statistical comparison was made by an analysis of variance (unpaired Student’s *t*-test).

## RESULTS

### TARGET PROTECTOR DESIGN AND *IN VITRO* TESTING

**FIGURE 1:**
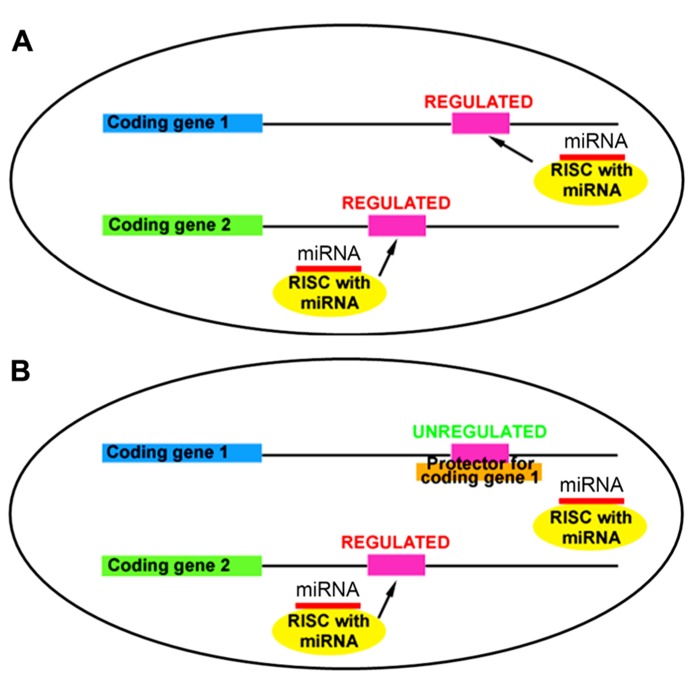
**Target protectors block miRNA action at a specific mRNA binding site. (A)** miRNAs regulate multiple targets through sequence-specific binding in the 3′UTR. **(B)** Target protectors block miRNA action at a single target, while other targets remain regulated.

In a typical cell, miRNAs target multiple mRNAs through partial sequence complementarity (**Figure 1A**). The concept behind target protectors is that a construct with perfect complementarity to a specific miRNA-binding site will outcompete the miRNA for binding at that site (**Figure 1B**). Thus, a single miRNA target is derepressed while the others remain regulated, allowing analysis of the effects of a single miRNA:mRNA relationship. To apply this concept in mammalian development, we designed plasmid-based mRNA target protectors using miR-19a:Pten regulation, since our previous work and others have demonstrated the targeting effect of miR-19a on Pten (Mu et al., 2009; Olive et al., 2009; Mavrakis et al., 2010; Bian et al., 2013).

**FIGURE 2:**
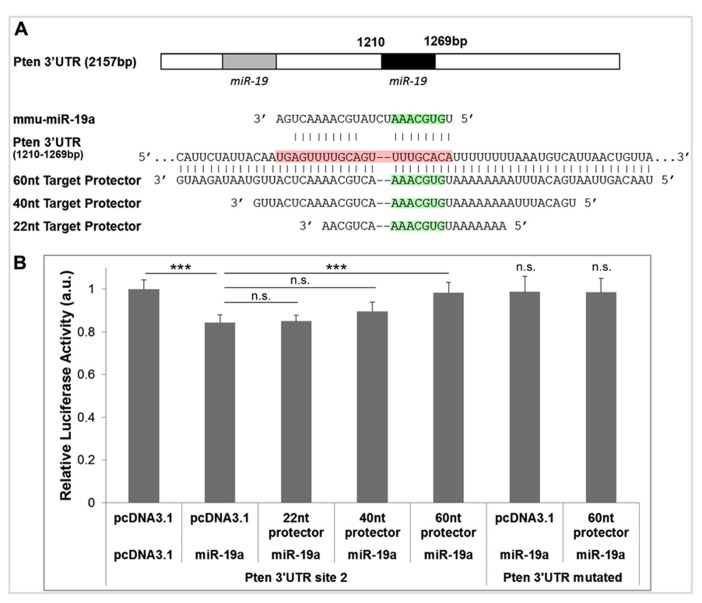
**Target protectors for *Pten* block miR-19a-induced repression. (A)** Binding sites of miR-19a in the *Pten* 3′UTR and complementary target protector sequences for the second miR-19a binding site. The seed binding sequence of miR-19a is highlighted in green, and the entire length of miR-19a along the *Pten* 3′UTR is highlighted in red. **(B)** Luciferase assays of target protector effects on miR-19a repression of the second miR-19a binding site in the *Pten* 3′UTR. miR-19a reduced luciferase activity in the absence of target protector. The 60 nt target protector but not the 22 or 40 nt target protector recovered luciferase activity of the *Pten* 3′UTR. Neither miR-19a nor the 60 nt target protector had an effect on the luciferase activity of the full length *Pten* 3′UTR when the miR-19a binding sites were mutated. Data are presented as mean ± SEM; *n* = 3 luciferase assays; *p* values in relation to control (***p < 0.01). n.s., not significant.

To optimize the minimum length of sequence complementarity necessary for maximum protector efficacy, we designed target protectors with three lengths of complementarity for the second miR-19a binding site in the *Pten* 3′UTR (**Figure 2A**). We chose the shortest length of 22 nt because this is the approximate size of most miRNAs (**Figure 2A**). We also designed protectors of 40 and 60 nt to increase complementarity, binding strength, and specificity (**Figure 2A**). Our basic rules for protector design are: (1) each protector is centered over the predicted miRNA seed binding site, (2) the protectors have perfect complementarity along the length of the 3′UTR, and (3) the protector must not overlap any other known functional sites in the 3′UTR. For the 22 and 40 nt target protectors, junk DNA sequences were inserted outside of the complementary region so that the overall length of all three constructs was constant with the 60 nt protector.

We hypothesized that an effective target protector would block miR-19a activity in the 3′UTR of *Pten* in a luciferase assay, resulting in a recovery of luciferase activity. To test the target protectors, we used a luciferase vector containing only the second miR-19a binding site of the *Pten* 3′UTR (the first miR-19a binding site was mutated in the *Pten* 3′UTR), and cotransfected it with miR-19a and each target protector in N2a cells. In the absence of any target protector, luciferase activity of the *Pten* 3′UTR was significantly decreased (**Figure 2B**). The 22 and 40 nt protectors did not show any significant recovery of luciferase activity, while the 60 nt protector showed a significant, almost complete recovery of activity (**Figure 2B**). Neither miR-19a nor any of the target protectors had an effect on the luciferase activity of the *Pten* 3′UTR containing a mutation in both miR-19a binding sites (**Figure 2B**). Our results indicate that the 60 nt target protector is the most effective at blocking miR-19a activity at the *Pten* 3′UTR

### 60 nt TARGET PROTECTOR BLOCKS miR-19a ACTIVITY *IN VITRO*

**FIGURE 3:**
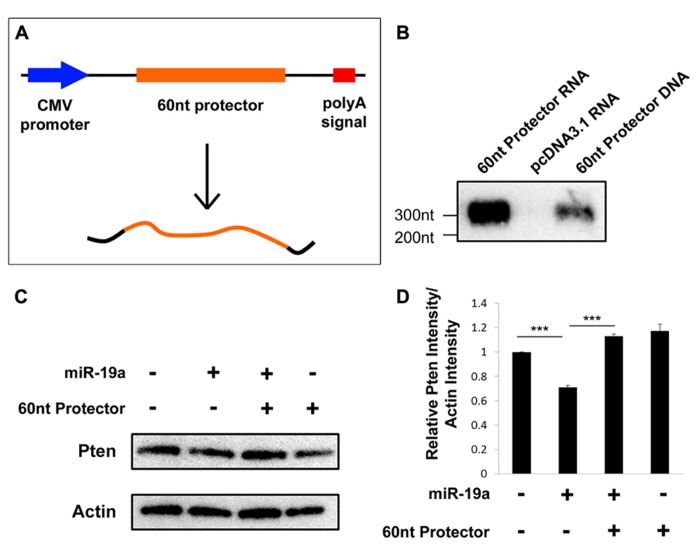
**Target protectors are transcribed and effective *in vitro*. (A)** Schematic vector map of the 60 nt target protector in pcDNA3.1. **(B)** The 60 nt target protector and control target protector DNA were detected by Northern blot assays at the expected size. No target protector was detected in RNA extracted from empty vector-transfected cells. **(C,D)** The endogenous protein levels of Pten were decreased upon transfection of miR-19a but recovered upon cotransfection of miR-19a and the 60 nt target protector. Transfection of the 60 nt target protector alone also showed an increase in Pten. Data are presented as mean ± SD; *n* = 3 separate protein extractions; *p* values in relation to control (***p < 0.001).

To ensure that the 60 nt target protector was transcribed and expressed as expected, we performed a northern blot assay using RNA extracted from N2a cells transfected with either the target protector or an empty vector. As a positive control, we also included digested vector DNA containing the protector. Based on the insert size and the predicted transcription start site and polyadenylation signals of the vector, the expect RNA size to be about 305 nt (**Figure 3A**). The protector is detected at the expected size in target protector transfected RNA and is not detected in the empty vector-transfected RNA, indicating that the protector is transcribed and expressed (**Figure 3B**).

We previously established that miR-19a regulates *Pten* posttranscriptionally by preventing its translation (Bian et al., 2013). Thus, we hypothesized that transfection of the protector should result in increased endogenous Pten protein in N2a cells. Indeed, a Western blot assay showed that transfection of only exogenous miR-19a results in a decrease of endogenous Pten, while cotransfection of exogenous miR-19a and the target protector, or the protector alone significantly rescues the endogenous Pten protein levels (**Figures 3C, D**). Our results suggest that the plasmid-based target protectors are transcribed and work to block posttranscriptional regulation by miRNAs.

### 60 nt TARGET PROTECTOR BLOCKS miR-19a ACTIVITY *IN VIVO*

**FIGURE 4:**
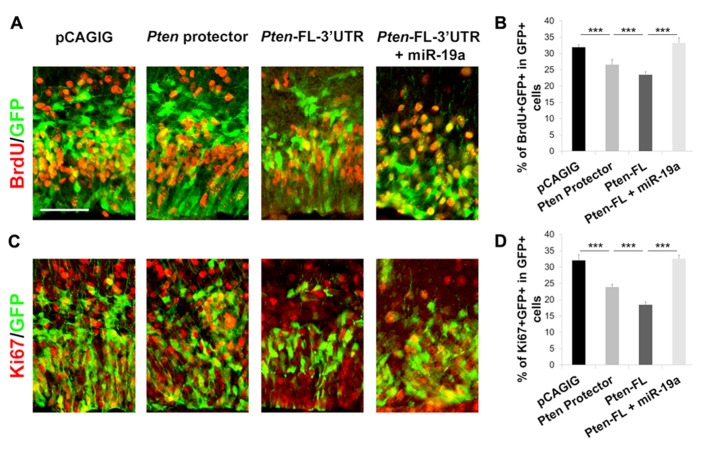
**Target protectors block miR-19a repression of *Pten* in the developing mouse cortex. (A–D)** Electroporation of the 60 nt target protector at E13.5 for analysis at E14.5 significantly decreased the number of BrdU-incorporating or Ki67^+^ proliferative cells colabeled with GFP in the cortex. Ectopic expression of full length *Pten* containing the 3′UTR (*Pten-FL-3*′*UTR*) showed a similar effect, while co-electroporation of *Pten-FL-3*′*UTR* and miR-19a ablated this effect. Data are presented as mean ± SD; *n* ≥ 3 for all electroporations; *p* values in relation to control (***p < 0.001). Scale bar: 50 μm.

Having established that the plasmid-based target protector is effective *in vitro*, we sought to apply it *in vivo*, where such a tool can provide insight to the function of specific miRNA–mRNA interactions during development. We previously established that miR-19a targeting of *Pten* promotes progenitor cell expansion in the developing mouse cortex (Bian et al., 2013). We expected that blocking miR-19a activity with the target protector will result in decreased proliferation of progenitors. To observe the maximum effect of blocking miR-19a binding to *Pten*, we also designed a 60 nt target protector for the first binding site in the *Pten* 3′UTR and used *in utero* electroporation to introduce protectors for both miR-19a binding sites in the *Pten* 3′UTR into the embryonic day 13.5 (E13.5) cortex, analyzed at E14.5. The numbers of BrdU^+^ and Ki67^+^ cells were significantly decreased after *Pten* target protectors were electroporated compared to empty vector electroporation, suggesting a functional result of the blockade of miR-19a silencing effect on Pten expression (**Figure 4**).

To test further whether decreased proliferation of neural progenitors in target protector electroporation is caused by up-regulation of Pten, which is usually suppressed by miR-19a, we electroporated full length *Pten* containing the 3′UTR with miR-19a binding sites (*Pten-FL-3*′*UTR*). We observed a significant decrease in both BrdU^+^ and Ki67^+^ cells upon electroporation of the *Pten* overexpression construct compared to empty vector electroporation (**Figures 4B, D**). When *Pten-FL-3*′*UTR* was co-electroporated with exogenous miR-19a, the numbers of BrdU^+^ and Ki67^+^ cells were recovered to wild type levels, indicating that the change is dependent on miR-19a activity on the *Pten* 3′UTR (**Figure 4**). Our results demonstrate that plasmid-based target protectors can be electroporated into developing cortices to block miRNA action at a specific target.

### TARGET PROTECTORS REVEAL THE ROLE OF miR-19a REPRESSION OF Pten IN THE DEVELOPING CORTEX

**FIGURE 5:**
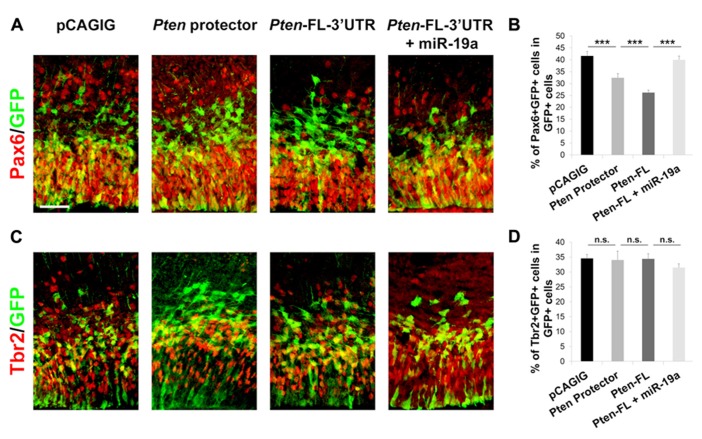
**miR-19a targeting of *Pten* is responsible for RGC expansion in the developing mouse cortex. (A,B)** miR-19a targeting of *Pten* is important for RGC expansion. Introduction of the 60 nt target protector at E13.5 for analysis at E14.5 significantly decreased the number of RGCs colabeled with GFP and Pax6. Ectopic expression of *Pten-FL-3*′*UTR* showed a similar effect, while co-electroporation of *Pten-FL-3*′*UTR* and miR-19a ablated this effect. **(C,D)** Ectopic expression of the 60 nt target protector, *Pten-FL-3*′*UTR*, or *Pten-FL-3*′*UTR* and miR-19a had no effect on the number of IPs colabeled with GFP and Tbr2. Data are presented as mean ± SEM; *n* ≥ 3 for all electroporations; *p* values in relation to control (***p < 0.001). n.s., not significant. Scale bar: 50 μm.

During corticogenesis, the transition of proliferative radial glia cells (RGCs) and intermediate progenitor (IP) into postmitotic neurons is tightly regulated in order to generate an appropriate amount of neurons while maintaining a progenitor pool. Our previous work has shown that the miRNA family miR-17-92, containing miR-19a, promotes RGC proliferation (Bian et al., 2013). To validate that miR-19a targeting of *Pten* is responsible for RGC proliferation, we introduced *Pten* target protectors into E13.5 cortices, which should result in a decrease of RGCs. We found that the number of Pax6^+^ RGCs is significantly decreased, while the number of Tbr2^+^ IPs is not changed (**Figure 5**).

To confirm further that *Pten* is responsible for the decrease of RGCs, we electroporated *Pten-FL-3*′*UTR*, which we expected to show a similar phenotype as the *Pten* target protectors, in E13.5 cortices collected at E14.5. Indeed, the number Pax6^+^ RGCs was decreased and the number of Tbr2^+^ IPs was not changed (**Figures 5B, D**). When *Pten-FL-3*′*UTR* and miR-19a were co-electroporated, the number of Pax6^+^ RGCs was recovered, while the number of Tbr2^+^ IPs remained unchanged (**Figure 5**). These results show that miR-19a targeting of *Pten* is critical for proliferation of RGCs in the developing cortex but does not affect the IP cell population. Using our *Pten* target protectors, we further demonstrate that the specific effects of miR-19a on RGC expansion occur through silencing *Pten*.

## DISCUSSION

miRNAs have been proven to play critical roles in development of invertebrates and vertebrates (Carrington and Ambros, 2003; Alvarez-Garcia and Miska, 2005; Kloosterman and Plasterk, 2006). Since one miRNA has multiple targets, it has been a daunting task to demonstrate which genes are major targets in specific cells or tissues during development. In this study, we have designed a plasmid-based tool for analyzing specific miRNA:target relationships, shown that it works effectively *in vitro* and *in vivo*, and applied it to a miRNA:mRNA pair in the developing mouse cortex.

In the past, many studies have examined the roles of individual miRNAs by removing them globally or conditionally. In *Drosophila* and mice, genetic mutants have been generated, while in other systems such as zebrafish or *Xenopus*, morpholinos or other antisense oligonucleotides have been used to knockdown miRNAs (Sokol, 2005; Karres et al., 2007; Ventura et al., 2008; Woltering and Durston, 2008; Conte et al., 2010; Rasmussen et al., 2010). miRNA sponges with multiple miRNA-binding sites have also been used to soak up mature miRNAs (Ebert et al., 2007; Loya et al., 2009; Ebert and Sharp, 2010). While these methods provide a genetic approach to explore the overall function of a miRNA, they lack the power to attribute any phenotype to a particular mRNA target. Later, morpholino-based target protectors were applied in zebrafish and *Xenopus* to examine the importance of miRNA repression of a specific target (Choi et al., 2007; Bonev et al., 2011; Stanton and Giraldez, 2011). While this technique has been useful in some model organisms, it is not applicable to many mammalian systems.

We have shown that plasmid-based target protectors are effective in mammalian systems. They are a simple and clean method for blocking miRNA action at a single target, eliminating uncertainty surrounding which mRNA targets are responsible for a phenotype. Because they are plasmid-based, these target protectors can be applied across a wide variety of model systems and tissues. They also have long lasting expression and can be used to study gene functions at any stage of development.

Here, we used *in utero* electroporation to introduce target protectors into the developing cortex; however, this is not the only application of plasmid-based target protectors. These protectors could also be used in viral transfection, or they could be incorporated to create transgenic lines. For any method of delivery, a conditional expression vector can be used to analyze a miRNA:target relationship in a specific cell type and at a specific stage. These target protectors also have disease treatment potential, as they could be used to increase expression of a dysregulated gene by blocking a miRNA.

While our plasmid-based target protectors are shown to be very effective for dissecting the miR-19a:*Pten* relationship, we have applied them to other miRNA:target pairs with mixed results. First, it is important to reiterate that these protectors are not applicable to every miRNA-binding site: if the protector overlaps another miRNA-binding site, protein binding site, or interferes with other RNA processing signals, then the results will be difficult to interpret. Another factor that may influence protector function is RNA secondary structure, either of the target RNA or the protector itself. To alleviate this problem, we recommend designing and testing target protectors of multiple lengths; though we found the 60 nt target protector to be the most effective, this may not be true in every case.

Another reason that a single target protector might not show a phenotype is that miRNAs often target multiple members of the same pathway. Thus, derepression of a single pathway member may be masked by continued regulation of the rest of the pathway. In this study, we successfully introduced target protectors for two miR-19a binding sites and we recommend this combinatorial approach for derepression of multiple pathway members. However, this method is limited by the maximum amount of DNA that can be introduced at a time. A large amount of each target protector is required to see an effect, which would be diluted by including multiple target protectors.

Plasmid-based target protectors open myriad opportunities in the miRNA field for dissecting specific miRNA:target interactions in mammalian model systems. These target protectors are applicable across tissues and developmental systems, and can be introduced in many ways. Here, we have presented basic concepts of target protector design and shown their application both *in vitro* and *in vivo*. We also revealed an important role of the miR-19a:*Pten* interaction in the developing cortex, demonstrating the great potential of this essential new tool.

## Conflict of Interest Statement

The authors declare that the research was conducted in the absence of any commercial or financial relationships that could be construed as a potential conflict of interest.
